# Predicting Patient-ventilator Asynchronies with Hidden Markov Models

**DOI:** 10.1038/s41598-018-36011-0

**Published:** 2018-12-04

**Authors:** Yaroslav Marchuk, Rudys Magrans, Bernat Sales, Jaume Montanya, Josefina López-Aguilar, Candelaria de Haro, Gemma Gomà, Carles Subirà, Rafael Fernández, Robert M. Kacmarek, Lluis Blanch

**Affiliations:** 1 Better Care S.L., Sabadell, Spain; 2 Critical Care Center, Parc Taulí Hospital Universitari, Institut d’Investigació i Innovació Parc Taulí, Universitat Autònoma de Barcelona, Sabadell, Spain; 3 Centro de Investigación Biomédica en Red de Enfermedades Respiratorias (CIBERES), Instituto de Salud Carlos III, Madrid, Spain; 4 Intensive Care Unit, Fundació Althaia, Universitat Internacional de Catalunya, Manresa, Spain; 5 Department of Respiratory Care, Department of Anesthesiology, Massachusetts General Hospital, Harvard Medical School, Boston, MA USA

## Abstract

In mechanical ventilation, it is paramount to ensure the patient’s ventilatory demand is met while minimizing asynchronies. We aimed to develop a model to predict the likelihood of asynchronies occurring. We analyzed 10,409,357 breaths from 51 critically ill patients who underwent mechanical ventilation >24 h. Patients were continuously monitored and common asynchronies were identified and regularly indexed. Based on discrete time-series data representing the total count of asynchronies, we defined four states or levels of risk of asynchronies, z1 (very-low-risk) – z4 (very-high-risk). A Poisson hidden Markov model was used to predict the probability of each level of risk occurring in the next period. Long periods with very few asynchronous events, and consequently very-low-risk, were more likely than periods with many events (state z4). States were persistent; large shifts of states were uncommon and most switches were to neighbouring states. Thus, patients entering states with a high number of asynchronies were very likely to continue in that state, which may have serious implications. This novel approach to dealing with patient-ventilator asynchrony is a first step in developing smart alarms to alert professionals to patients entering high-risk states so they can consider actions to improve patient-ventilator interaction.

## Introduction

Patients in intensive care units (ICU) sometimes need mechanical ventilation to improve alveolar ventilation and oxygenation while decreasing the load on the respiratory muscles. Patients may undergo mechanical ventilation for several days until their condition improves. Although mechanical ventilation is a life-saving intervention, numerous complications can develop. Ventilator cycles must match the patient’s own rhythm of breathing; however, mismatching is common, resulting in poor patient-ventilator interaction with deleterious consequences^1–5^. When patient-ventilator asynchronies occur, breathing becomes more difficult and the patient’s condition can worsen. Asynchronies are more dangerous when their frequency is relatively high^4^. Asynchronies can prolong mechanical ventilation and ICU stays^4,6^, increase the probability of respiratory muscle and lung injury^7,8^, increase mortality^3,9^, and lead to other complications^10,11^.

Personalized or precision medicine is an emerging concept that will change clinical practice in ICUs in the short-to-mid term, helping physicians choose the right therapy at the right time^12,13^. ICU patients are intensely and continuously monitored, generating extremely large datasets. All this data is readily available and can be exploited with big data tools and automated learning systems, providing a unique opportunity to improve decision-making in this demanding environment.

Based on their understanding of the physiological principles involved and evidence from clinical studies, physicians manage mechanical ventilation by assessing waveforms on bedside monitors. However, most perform poorly at managing patient-ventilator interactions, often failing to identify common forms of asynchronies^14,15^. Moreover, patients take several thousands of breaths each day, and busy professionals can observe only a small proportion of these. Early detection of an increased frequency of asynchronies could alert physicians to immediately diagnose and manage the problem. Although new technologies enable continuous bedside monitoring to detect asynchronies automatically^3,16–19^, monitoring systems are as yet unable to predict asynchronies in real time.

Time series forecasting aims to predict future events based on past observations. A hidden Markov model (HMM)^20,21^ is a kind of statistical approach applied in data mining for recognizing patterns over time. Sequential data can be represented as a Markov chain of latent (or hidden) states with observable, state-dependent output (in the form of data points). HMM have proven useful in a variety of fields, such as computational biology^22^, physiological time series analysis^23,24^, speech recognition^25^, financial problems^26^, and others^27^. Currently, the incidence of asynchronies is classified as low or high with an arbitrary cut-off of 10%^4,28^. In the setting of this study, the hidden states can be interpreted as proxies for patients’ level of synchrony with the ventilator; each state can be associated with a different frequency of events and therefore results in a different level of risk.

We aimed to obtain proof of concept that regularly indexing the most common types of patient-ventilator asynchronies along time can generate a discrete time series that can be used to predict the probability of asynchronies occurring in future periods. To this end, we employed an HMM. To take into account the wide heterogeneity among ICU patients and high complexity of critical illness, we considered time series covering the entire period of mechanical ventilation in patients with different conditions. Although not a specific aim of this study, we also conducted a subanalysis of the probable effects of asynchronies on some cardiovascular parameters.

## Methods

### Patients and data

Patients admitted to rooms equipped with the Better Care^®^ system (Better Care SL, Spain) in one of two ICUs in the period comprising September 2011 through May 2016 were potentially eligible. Exclusion criteria were age <18 years, mechanical ventilation for <24 hours, pregnancy, do-not-resuscitate orders, chest tubes with suspected bronchopleural fistula, and admission for organ donation. Finally, 51 patients who met the above criteria were selected.

The Comitè d’Ètica d’Investigació amb medicaments at the Corporació Sanitària Parc Taulí and the Clinical Research Ethics Committee of Fundació Unió Catalana d’Hospitals approved the study and waived informed consent because the study was observational, posed no added risk to the patient, and did not interfere with usual care.

ICU rooms were equipped with one of the following ventilators: Evita 4 (Dräger, Germany), Puritan Bennet 840 (Covidien, US), or Servo I (Maquet, Sweden). Better Care^®^ uses drivers specifically designed to continuously capture output signals from different bedside medical devices (ventilators, multiparameter monitors, etc.)^3,16,19^. These signals are resampled at 200 Hz and processed with dedicated algorithms to obtain a set of physiological variables and time events. From the airway pressure and airflow signals, the system detects the most common types of asynchronies (ineffective inspiratory efforts, double cycling, short cycling, and prolonged cycling). See the Supplementary Methods and the Supplementary Fig. S1 for definitions of each asynchrony. For each breath, the system determines whether one or more asynchrony events are present and stores this information in a PostgreSQL (Berkeley, CA; https://www.postgresql.org/) database for further analyses.

Sampling at regular periods of time (T), we generated time series representing the number of events (i.e., the asynchronies listed above) that occurred in each period. In this study, respiratory cycles were grouped in periods of 5, 10, 15, 20 and 25 minutes, and asynchrony events occurring in each of these time intervals were counted throughout recording.

**Figure 1:**
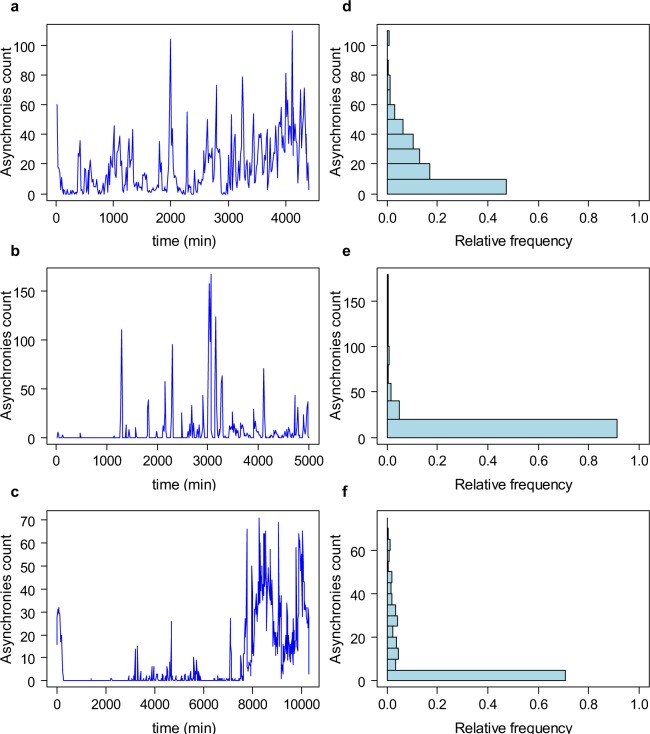
Time series of asynchronies count indexed each 15 min (**a**–**c**) and their corresponding histograms (**d**–**f**) from three representative patients. Note these are discrete time series of non-negative values, and positively skewed (histogram characterized by a long tail in the positive direction) with several observations having a value close to zero, which justifies the use of a Poisson distribution to model this kind of data.

Figure 1 shows representative examples of the evolution of the number of events and their distributions in three patients.

### Algorithm

Because HMMs can detect states with different frequencies of events, they may be able to predict the number of asynchrony events that will occur. HMMs automatically detect whether a patient is in a ‘low-risk state’ (i.e., low frequency of asynchrony events) or in a ‘high-risk state’ (i.e., high frequency of asynchrony events). The number of states is a parameter that needs to be set by the user before training the model. Given any number of possible predefined states, the model finds the most probable distribution for each state, a posteriori, and also makes it possible to detect when the patient changes from one state to another. Then, the uncertainty of being in each state, represented by this posterior probability distribution, can be summarized in terms of credible intervals.

According to previous investigations^3,29^, the model was estimated with four states, z1–z4, that would match usual clinical judgement at the bedside. The state with the lowest number of events is z1 (very-low-risk state), representing good interaction with the ventilator with almost no asynchronies; states z2 and z3 are intermediate states (low-risk and high-risk states, respectively) with increasing mismatch between the patient and the ventilator; and z4 is the state with the highest number of events (very-high-risk state), where asynchrony is severe because the incidence of events is high and might lead to considerable patient distress and might increase the risk of ventilator-associated lung injury.

We used a Poisson distribution for the emission probabilities associated with each state (also known as output probabilities). Therefore, the HMM consisted of a Poisson regression with intercept only and the patient’s state as a categorical variable. The model and the predictions were built using time intervals of 5, 10, 15, 20, and 25 minutes. The predictions are one-step ahead forecasts of the number of events. The expectation maximization (EM) algorithm iteratively computes the transition and emission probabilities^21^; the EM algorithm initializes with random values for the transition and emission probabilities. Next, the Viterbi algorithm^30^ uses the emission and transition probabilities estimated earlier to find the most likely sequence of latent states (i.e., the posterior probability distribution) that generated the data. See the Supplementary Methods for more specific details about HMM.

In addition to the predictions of the expected counts, we also made predictions of the expected rate of asynchrony events (i.e., number of event counts divided by the total number of respiratory cycles per period of observation T) to obtain values that would be easier to interpret clinically. This step used a generalized linear model with the parameter obtained from the fitted HMM. See the Supplementary Methods for additional information.

We used R 3.3.1 (R Core Team, Vienna, Austria, URL http://www.R-project.org/) with the RPostgreSQL package (Berkeley, CA; https://CRAN.R-project.org/package=PostgreSQL) to interface with the database and the depmixS4 package^31^ to fit and analyze the HMM models.

### Analysis of cardiovascular parameters

From the posterior probability distributions, summarized in terms of credible intervals, we identified periods of asynchronies belonging to each state and matched them with the corresponding periods in the cardiovascular time series (heart rate and oxygen saturation time series) sampled every 15 minutes. Then, we used descriptive statistics to analyze the mean heart rate in beats per minute (bpm), the mean level of oxygen saturation (%), and episodes of bradycardia, tachycardia, and hypoxemia.

## Results

Patients’ baseline characteristics (Supplementary Table S1) are reported as medians (25^th^, 75^th^ percentiles) for continuous variables, unless otherwise specified. We analyzed a total of 10,409,357 breaths from the 51 patients. To test the model’s ability to predict new data and to estimate the model’s generalizability, we used a k-fold cross validation (k = 5) procedure. This validation procedure was also used to test the fit of the HMM with only intercept versus a base model whose prediction is always zero events, as this is the most likely outcome. The final model was constructed on the total sample of patients.

**Figure 2:**
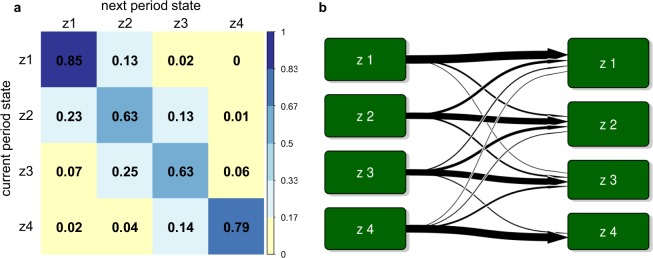
State transition for the Poisson hidden Markov models from the time series indexed each 15 min. (**a**) Transition probability matrix. Values in each cell represent average probability computed on the total sample of patients. Diagonal of the matrix represents the probabilities of not changing in the next period. Cells with zero probability represent a value < 0.005. (**b**) State transition diagram. Arrows indicate the probability of transition from each state to other ones: thicker arrows indicate higher probability; no arrow indicates the probability is zero (or near zero).

Figure 2 shows the transition probabilities of the model for time intervals of 15 minutes. The probability of switching from one state to another decreased with the distance between states, being 0.13 for the switch from z1 to z2, 0.02 for the switch from z1 to z3, and <0.005 for the switch from z1 to z4. The probability values of the diagonal were large, signifying that all four states were highly persistent. Thus, the state at period *t* + 1 is very likely to be the same as the state at period $t$. Analyzing time series defined for the other time intervals yielded similar results (see Supplementary Fig. S2).

**Figure 3:**
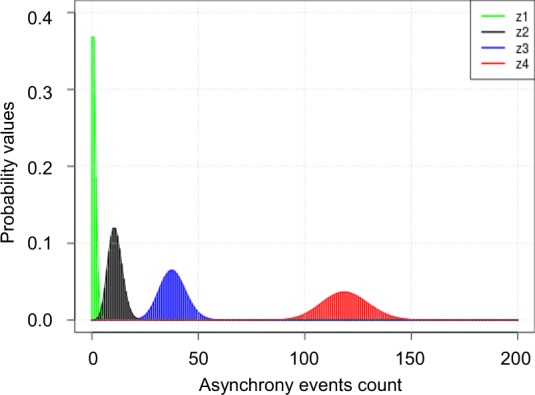
Emission probabilities for each state (z1–z4) in a 15-minute interval. Note state z1 (i.e., that with the lowest number of events and consequently lowest level of risk) is the most likely, whereas state z4 (i.e., that with the highest level of risk) is the less likely.

The probability distributions of the observations for the four states were computed with only intercept. This means that there is a unique probability distribution for each state. Figure 3 shows the probability distribution for each of the four states for a time interval of 15 minutes. Table 1 shows the average number of events expected to occur in each state and the proportion of time that patients spent in each state. Patients spent 52% of the time in the lowest state, z1, where a mean (95% CI) of asynchrony event expected to occur was 1 (0, 3). By contrast, patients spent 14% of the time in z3 (where a mean of 38 (26, 51) asynchrony events occurred) and 6% of the time in z4 (where a mean number of 119 (98, 141) asynchrony events occurred). The approximate percentage of asynchrony events (Table 1) is 11.6 (11.6, 11.7) in z3 and 35.1 (35, 35.3) in z4. See Supplementary Table S2 for the analyses with the other time intervals.

**Table 1:** Mean (95% CI) expected number (*λ*) of asynchrony events for time series defined each T = 15 minutes, approximate expected rate determined by a generalized linear model, and time spent in each state (tspent) represented as a proportion of the total time.

Variable	Values for each state			
	z1	z2	z3	z4
*λ*	1 (0, 3)	11 (5, 18)	38 (26, 51)	119 (98, 141)
rate (%)	0.2 (0.19, 0.2)	3.38(3.36, 3.39)	11.6(11.6, 11.7)	35.1(35.0, 35.3)
tspent	0.52	0.28	0.14	0.06

To assess the accuracy of the models, we computed the root mean square error (RMSE) for the HMM with only intercept and the base model. This step was conducted by means of k-fold cross validation on five different training and validation subsets. The HMM’s predictions with only intercept were better, RMSE(mean and (standard error)) = 19.5 (3.12), than the base model’s predictions, RMSE = 35.4 (6.58).

Descriptive information about the behaviour of some cardiovascular parameters in each state is reported in Table 2 and Supplementary Fig. S3. Oxygen saturation (Supplementary Fig. S3) seemed to decrease during state z4, and consequently, both the frequency and duration of hypoxemia episodes (Table 2) seemed to increase. By contrast, the heart rate and bradycardia and tachycardia episodes were similar in the different states.

**Table 2:** Characterization of some cardiovascular episodes during asynchronies, by state. Percentage of 15-minute periods (%episode) with at least one episode of bradycardia, tachycardia, and/or hypoxemia, with respect to the total number of T = 15-minute periods; and mean (SD) percentage of time within each T = 15-minute periods (tepisode) with episodes of tachycardia, bradycardia, and/or hypoxemia.

Episodes	Values for each state			
	z1	z2	z3	z4
**Bradycardia**				
%episode	8.36	7.74	4.55	9.07
tepisode	4.42 (18.8)	2.43 (12.9)	1.11 (8.58)	1.86 (11.7)
**Tachycardia**				
%episode	39.3	42.4	46.3	36
tepisode	25.5 (40.9)	22.9 (38.3)	28.2 (40.9)	26.9 (42.6)
**Hypoxemia**				
%episode	5.94	11.5	16.1	15.1
tepisode	1.95 (12.2)	2.51 (12.6)	3.66 (14.9)	5.13 (19.4)

Bradycardia episode = heart rate <60 bpm; Tachycardia episode = heart rate >100 bpm; Hypoxemia episode = oxygen saturation <90%.

## Discussion

This proof-of-concept study shows that it is feasible to use HMMs to predict patient-ventilator asynchronies in critically ill patients and to infer the probability that the number of asynchrony events will be above a given threshold. Previous studies^3,14,16,32,33^ aimed to detect asynchronies once they occurred; to our knowledge, this is the first study focused on predicting asynchrony events before they happen. Unlike other studies based on very limited observation periods in narrowly defined subpopulations of patients with specific conditions^7,28^, we analyzed the entire period of mechanical ventilation in a heterogeneous population of ICU patients with a wide variety of critical illnesses, increasing the generalizability of the results and the model’s ability to take trends into account.

The HMM was able to detect states with different levels of risk depending on the expected count of asynchrony events. Patients spent long periods of time with very few events and short periods of time with many events. Large shifts between states were unlikely, as the most probable switches were to “neighbouring” states. Patients tended to remain in the same state even when large intervals of time were considered. This implies that once patients entered a state with a high number of asynchronies, they would very likely continue in that state in the following periods, increasing the likelihood of serious complications.

The intensity of the asynchronies probably has more prognostic importance than the overall average occurrence^34^. Vaporidi *et al*.^9^ recently found that although the overall rate of ineffective inspiratory efforts as a percentage of total breaths was not associated with outcome even when it was greater than 10%, clusters of ineffective efforts were associated with prolonged MV and increased mortality, suggesting a dose effect. Similarly, double cycling can also occur in clusters, although the clinical importance of clusters of double-cycling events remains to be characterized^29^. Therefore, although the states with higher numbers of asynchronies do not last long, they are likely to have more clusters of asynchronies.

How long patients tolerate poor interaction with the ventilator is unknown. Vaporidi *et al*.^9^ considered periods of ineffective efforts lasting more than 3 min to be clusters, although the median duration of the clusters ranged from 23 min to 17 min during their 6-day follow-up period. Similarly, de Haro *et al*.^29^, defining clusters of double cycling as periods in which at least 10% all breaths in a 3-min period were double cycled, found that the median duration of clusters was 15.5 min. Thus, we decided that a 15-min interval would be a reasonable period for physicians to deal with asynchronies. However, we also analysed other intervals (results in supplementary material).

Patient-ventilator asynchrony can lead to considerable patient distress, and it also impedes the effectiveness of the ventilator in decreasing work of breathing or providing adequate alveolar ventilation, which may lead to an episode of acute hypoxemia^35^. Interestingly, our subanalysis found that more and longer-lasting episodes of low oxygen saturation levels occurred in z4 states, suggesting that periods of severe dyssynchrony between the patient and the ventilator might constitute a medical emergency.

HMMs could be used to create a system that could predict the likelihood of asynchrony events and alert professionals to the danger of clusters of asynchronies developing. Alarms could be set to go off when the predicted number of events exceeded a certain threshold or when the patient has entered in a state where a large number of events are expected and the possibility of clusters is greater. Such a tool would aid medical decision making, allowing staff to take actions (if necessary) to improve patient-ventilator interaction and to avoid complications from poor interaction. The implementation of such a system in routine daily care is beyond the scope of this proof-of-concept study; to guarantee reasonable performance, this approach must be validated with data from multiple ICUs to ensure that our results can be generalized.

Our computations of the HMM could not model the expected rate of the occurrence of events; rather they merely modelled the counts. To a certain extent, this technical shortcoming was overcome when making the predictions taking into account the total number of respiratory cycles per period of observation. This allowed us to express the average number of events in terms of a rate, providing an approximate value to illustrate the magnitude of the problem from a clinical standpoint.

Although HMMs seem feasible in this setting, other statistical models or machine-learning algorithms could also be used to predict asynchronies. Furthermore, additional physiologic variables (e.g., respiratory rate, inspiratory time, positive end-expiratory pressure, etc.) and ventilatory parameters (ventilation mode, tidal volume/peak pressure, etc.) could improve the accuracy of the predictions.

In summary, HMMs can predict periods with high frequencies of asynchrony events and could be used in an early-warning system. This study represents another step towards precision medicine in the ICU, which could lead to a more individualized ventilatory strategies and, consequently, better clinical outcomes and patient experiences.

## Electronic supplementary material

Electronic Supplementary Material

## Data Availability

The datasets generated during and/or analyzed during the current study are available from the corresponding author on reasonable request.
