# Supplementary material for: Predicting Patient-ventilator Asynchronies with Hidden Markov Models
Source: Sci Rep. 2018 Dec 4;8:17614. doi: 10.1038/s41598-018-36011-0 (PMC6279839; doi:10.1038/s41598-018-36011-0)
Supplement: Supplementary file 1 — Electronic Supplementary Material [file 41598_2018_36011_MOESM1_ESM.pdf]

Electronic Supplementary Material

Predicting Patient-ventilator Asynchronies with Hidden Markov Models

Yaroslav Marchuk[1]*, Rudys Magrans[2,3]*, Bernat Sales[1], Jaume Montanya[1], Josefina López-Aguilar[2,3], Candelaria de Haro[2,3], Gemma Gomà[2], Carles Subirà[4], Rafael Fernández[3,4], Robert M Kacmarek[5], Lluis Blanch[2,3]

* These authors contributed equally to this work.

[1]Better Care S.L., Sabadell, Spain.

[2]Critical Care Center, Parc Taulí Hospital Universitari, Institut d'Investigació i Innovació Parc Taulí, Universitat Autònoma de Barcelona. Sabadell, Spain

[3]Centro de Investigación Biomédica en Red de Enfermedades Respiratorias (CIBERES), Instituto de Salud Carlos III. Madrid, Spain

[4]Intensive Care Unit, Fundació Althaia, Universitat Internacional de Catalunya. Manresa, Spain

[5]Department of Respiratory Care, Department of Anesthesiology, Massachusetts General Hospital, Harvard Medical School. Boston, MA, USA

Contents

Supplementary Methods

- Definition of patient-ventilator asynchrony and different types
- Description of the algorithm based on hidden Markov model

Supplementary Tables S1-S2

Supplementary Figures S1-S3

Supplementary References

Supplementary Methods

Definition of patient-ventilator asynchrony and different types

Briefly, patient-ventilator asynchrony occurs when the phases of breath delivered by the ventilator do not match those of the patient. To meet the patient's demands, the ventilator's inspiratory time and gas delivery must match the patient's neural inspiratory time[1]. There are different types of asynchronies. Among the most prevalent are ineffective efforts, double cycling, short cycling, and prolonged cycling (see Supplementary Fig. S1).

Ineffective efforts are contractions of the inspiratory muscles, primarily the diaphragm, that are not followed by a ventilator breath. This asynchrony occurs when the pressure resulting from the patient's attempt to initiate a breath does not reach the ventilator's trigger threshold. In other words, the ventilator fails to detect the patient's inspiratory efforts, which are reflected physiologically by an increase in transdiaphragmatic pressure (decrease in esophageal pressure, increase in gastric pressure) and/or electrical activity of the diaphragm[2,3]. Ineffective efforts result in the patient's respiratory rate being higher than the ventilator's rate.

Double cycling consists of a sustained inspiratory effort that persists beyond the ventilator's inspiratory time, cessation of inspiratory flow, or the beginning of mechanical expiration, triggering a second ventilator breath, which may or may not be followed by a short expiration, where all or part of the volume of the first breath is added to the second breath[4]. Double cycling can cause ventilator-induced lung injury[5–7].

Short cycling occurs when the inspiratory time is less than one-half the mean inspiratory time, and prolonged cycling occurs when the inspiratory time is greater than twice the mean inspiratory time[4]. Inspiratory time is defined as the time during which gas flow is positive, and the mean inspiratory time is calculated over 20 cycles.

Description of the hidden Markov model

Next is a brief description of the hidden Markov model (HMM) used in our study. More general and specific details about this kind of statistical models can be found in Bishop (2007)[8].

Sequential data can be represented as a Markov chain of latent variables, with each observation conditioned on the state of the corresponding latent variable. An HMM assumes that observations are generated by different probability distributions corresponding to the discrete multinomial latent variable. In the setting of this study, the hidden states can be interpreted as proxies for patients' level of synchrony with the ventilator; each state can be associated with a different frequency of events and therefore results in a different level of risk.

Thus, HMM can detect states with different frequencies of events, so it can predict the number of events that will occur in a period. HMM automatically detects whether a patient is at a 'low-risk state' (low frequency of events) or at a 'high-risk state' (high frequency of events). The number of states is a parameter that needs to be set by the user before training the model. Given any number of possible predefined states, the model finds the most probable distribution for each state, a posteriori, and also makes it possible to detect when the patient changes from one state to another. Then, the uncertainty of being in each state, represented by this posterior probability distribution, can be summarized in terms of credible intervals.

The hidden Markov model

In the context of an HMM, x_t is defined as the number of events during the period t and z_t is the state associated with that period. There are k states, each of which has different characteristics. At period t, all states have a probability of occurring, but only one of them actually occurs.

An HMM has two main components: transition probabilities and emission probabilities.

Transition probabilities

The relationship between period t and period $t+1$ in the HMM is governed by the transition matrix, A, which represents the probability of switching from one state to another. If there are k states, A has dimension $k \times k$ and its elements $A[i,j]$ represent the probability of switching from state i to state j. Since the elements of the matrix are probabilities, their values must be between zero and one, and the sum of each row in the matrix must equal 1.

If the diagonal elements in A are much larger than the off-diagonal elements, then the data sequence will have long runs of points generated from a single state and infrequent transitions from one state to another; if the diagonal and off-diagonal elements are similar, the state of the sequence will change frequently. Thus, the values of A determine how persistent the states are.

Emission probabilities

The distribution of the observation x_t given the state is called the emission probability, denoted as $P(x_t|z_t, \phi_k)$. The different k hidden states are associated with different probability distributions with different parameters ϕ_k. At period *t*, the observation x_t is generated by one of the *k* possible probability distributions, depending on the state of that period z_t and the set of parameters ϕ_k that determine the chosen probability ditribution. In this study, the chosen probability distribution was the Poisson distribution, which is the most common choice for event counts[9]. Thus, ϕ_k is the parameter λ of the Poisson distribution, which represents the expected count of events at the state *k*. Therefore, if the model consists of k different states, the emission probabilities of those states are Poisson distributions with different parameters λ.

In HMM the data generating process works as follows: At period t, the hidden state has a certain probability of taking on any of the k possible predefined states, and this probability depends on the state in period $t-1$. These probabilities come from the corresponding values in the transition matrix A. Once a state is achieved, the observation x_t is sampled from that state's probability distribution, which is the emission probability. Then, at period $t+1$, the probabilities of the possible states depend on the state that was achieved in period t. Once the state z_{t+1} is achieved, the observation x_{t+1} is sampled from the distribution associated with that state. This process continues indefinitely until the last period. The first state of the process, given that it is does not have any previous state, is usually sampled from a distribution where all states have equal probability.

Estimation

The expectation maximization algorithm iteratively computes the transition and emission probabilities[10]. We initialize this algorithm with random values for the transition and emission probabilities. Next, the Viterbi algorithm[11] uses the emission and transition probabilities estimated earlier to find the most likely sequence of the latent states (i.e., the posterior probability distribution) that generated the data.

In many applications of hidden Markov models, the latent variables have some meaningful interpretation, and so it is often of interest to find the most probable sequence of hidden states for a given observation sequence. However, finding the most probable sequence of latent states is not the same as finding the set of states that are individually the most probable. The set of states that are individually the most probable will not correspond to the most probable sequence of states, because the sequence also depends on the transition probabilities. In fact, if the two successive states that are the most probable individually are connected by a transition matrix element whose value is zero, the probability of the sequence will be zero.

Prediction

At any period t, once the state is achieved, it is possible to predict the expected value of x_{t+1} from the estimates of the transition and emission probabilities. The prediction of x_{t+1} is a combination of all emission probability distributions weighted by the transition probabilities.

The model and the predictions were built using time intervals of 5, 10, 15, 20, and 25 minutes. The predictions are one-step ahead forecasts of the number of events. Therefore, a one-step ahead forecast with the model using time intervals of 15 minutes is the number of events predicted to occur in the next 15 minutes.

From a clinical standpoint, it is more meaningful to model the rate of asynchrony events than it is to model the counts. This rate would represent the number of events divided by the total number of respiratory cycles per period of observation. Unfortunately, technical limitations meant that the current model estimated only the expected counts.

To a certain extent, this shortcoming is overcome by using a generalised linear model (GLM) to make predictions. The GLM uses the parameters estimated by the hidden Markov model together with the number of respiratory cycles as an exposure variable to indicate the number of times the events could have happened. The exposure variable is set to the GLM in a logarithmic form, since time series events follow a Poisson distribution. This enables to make predictions in terms of the expected rate of the occurrence of events rather than merely the counts. See the Results in Table 1 (main text) and Supplementary Table S2.

Validation

To validate the model, we used a k-fold cross validation procedure. Five different training/validation subsets were randomly selected, so that all patients were used for both training and validation, and each patient was used just once for validation. Due to

the amount of data and the complexity of the algorithm, the number of folds is limited by computational power. Following this limitation, the selected number of folds was set to five. The model’s predictive ability was assessed in terms of a root mean squared error.

Characteristic	Value
Patients	n = 51
Age (years)	63 (54, 76)
Sex (male), n (%)	34 (66.7%)
Reason for MV, n (%)	
Acute respiratory failure	36 (70.6%)
Sepsis	13 (25.5%)
Pneumonia	8 (15.7%)
Acute respiratory distress syndrome	4 (7.8%)
Chronic obstructive pulmonary disease	4 (7.8%)
Congestive heart failure	2 (3.9%)
Other	5 (9.8%)
Postsurgical	6 (11.8%)
Neurologic	4 (7.8%)
Multiple trauma	3 (5.9%)
Cardiac arrest	2 (3.9%)
APACHE II	16.5 (11.3, 23)
SOFA at admission	7.5 (6, 10)
Length of MV (days)	6.5 (4, 10)
ICU stay (days)	11 (7, 19)
ICU mortality, n (%)	9 (17.7%)
Number of breaths ($x10^3$)	167.8 (101.9, 225.0)
Asynchrony count ($x10^3$)	5.56 (2.48, 9.97)

Supplementary Table S1. Patients characteristics. Data expressed as medians (25th, 75th percentiles) or percentages. APACHE II, Acute Physiology And Chronic Health Evaluation II score; SOFA, Sequential Organ Failure Assessment score.

Period T	Variable	Values for each state			
		z1	z2	z3	z4
5min	λ	0 (0, 0)	4 (1, 8)	14 (7, 22)	42 (30, 55)
	rate (%)	0.16 (0.16, 0.16)	3.57 (3.54, 3.59)	12.8 (12.7, 12.8)	37.5 (37.3, 37.6)
	tspent	0.57	0.25	0.13	0.05
10min	λ	0 (0, 0)	7 (2, 13)	25 (16, 35)	79 (62, 97)
	rate (%)	0.17 (0.17, 0.18)	3.39 (3.37, 3.41)	11.6 (11.6, 11.7)	35.2 (35.1, 35.3)
	tspent	0.54	0.26	0.14	0.06
20min	λ	1 (0, 3)	13 (6, 21)	47 (34, 61)	152 (128, 177)
	rate (%)	0.2 (0.2, 0.21)	3.15 (3.13, 3.17)	11.0 (10.9, 11.0)	34.1 (33.9, 34.2)
	tspent	0.5	0.29	0.15	0.06
25min	λ	1 (0, 3)	14 (7, 22)	54 (40, 69)	180 (154, 207)
	rate (%)	0.23 (0.23, 0.24)	3.49 (3.47, 3.51)	11.7 (11.6, 11.7)	33.8 (33.6, 33.9)
	tspent	0.52	0.3	0.14	0.05

Supplementary Table S2. Mean (95% CI) expected number λ of asynchrony events for time series defined each T = 5, 10, 20, and 25 minutes, approximate expected rate determined by a generalised linear model, and spent time in each state represented as a proportion of the total time.

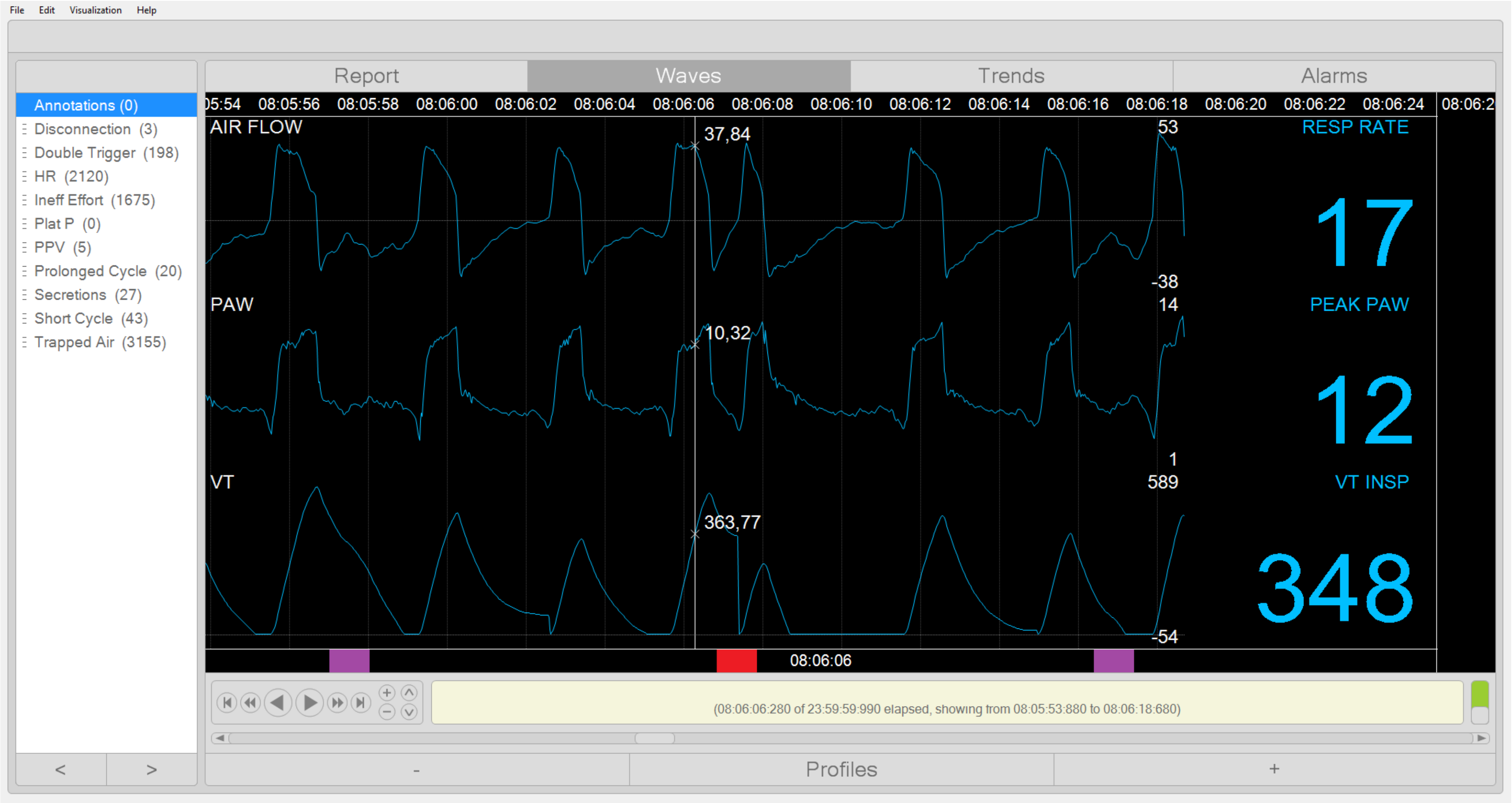

Supplementary Figure S1. Some common forms of patient-ventilator asynchronies. Airflow, airway pressure, and volume tracing where episodes of double cycling (red marks) and ineffective efforts (purple marks) were identified by Better Care™ software.

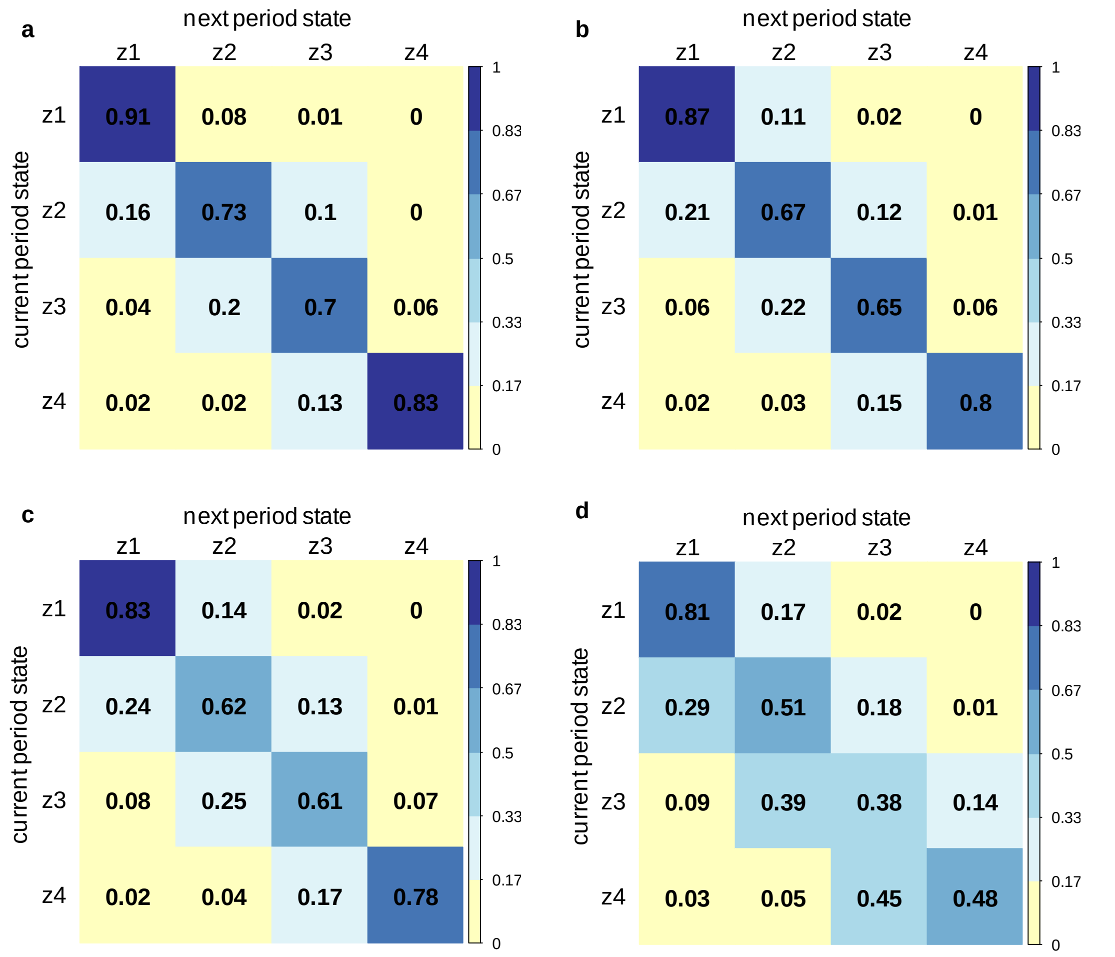

Supplementary Figure S2. Transition probability matrices of the Poisson hidden Markov models from the time series indexed each (a) 5min, (b) 10min, (c) 20min, and (d) 25 min. Values in each cell represent mean probability computed on the total sample of patients. Diagonal of the matrix represents the probability of not changing states in the next period. Cells with zero probability represent a value < 0.005.

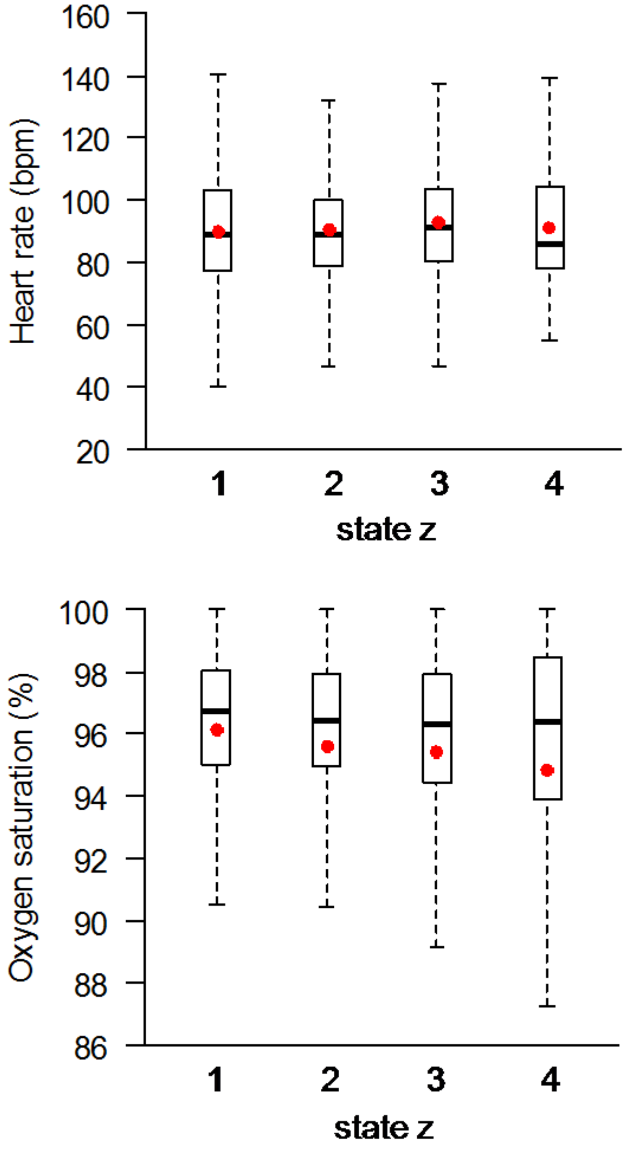

Supplementary Figure S3. Descriptive boxplots for the mean heart rate and the mean oxygen saturation each 15 minutes, by each state. Red dots represent means, and boxplots indicate medians and 25th-75th percentiles. Note that outliers have been omitted to facilitate visualization.

Supplementary References

1. Subirà, C., de Haro, C., Magrans, R., Fernández, R. & Blanch, L. Minimizing asynchronies in mechanical ventilation: current and future trends. *Respiratory Care* **63,** 464–478 (2018).
2. Georgopoulos, D., Prinianakis, G. & Kondili, E. Bedside waveforms interpretation as a tool to identify patient-ventilator asynchronies. *Intensive Care Medicine* **32,** 34–47 (2006).
3. Blanch, L. *et al.* Validation of the Better Care® system to detect ineffective efforts during expiration in mechanically ventilated patients: a pilot study. *Intensive Care Medicine* **38,** 772–780 (2012).
4. Thille, A. W., Rodriguez, P., Cabello, B., Lellouche, F. & Brochard, L. Patient-ventilator asynchrony during assisted mechanical ventilation. *Intensive Care Medicine* **32,** 1515–1522 (2006).
5. Figueroa-Casas, J. B. & Montoya, R. Effect of tidal volume size and its delivery mode on patient–ventilator dyssynchrony. *Annals of the American Thoracic Society* **13,** 2207–2214 (2016).
6. Gattinoni, L. *et al.* Ventilator-related causes of lung injury: the mechanical power. *Intensive Care Medicine* **42,** 1567–1575 (2016).
7. Pohlman, M. C. *et al.* Excessive tidal volume from breath stacking during lung-protective ventilation for acute lung injury. *Crit. Care Med.* **36,** 3019–3023 (2008).
8. Bishop, C. M. *Pattern recognition and machine learning*. (Springer, 2006).
9. Cameron, A. C. & Trivedi, P. K. *Regression analysis of count data*. (Cambridge University Press, 2013).
10. Baum, L. E., Petrie, T., Soules, G. & Weiss, N. A maximization technique occurring in the statistical analysis of probabilistic functions of Markov chains. *The Annals of Mathematical Statistics* **41,** 164–171 (1970).
11. Viterbi, A. Error bounds for convolutional codes and an asymptotically optimum decoding algorithm. *IEEE Transactions on Information Theory* **13,** 260–269 (1967).
